# Risk factors for delayed neuro-surgical intervention in patients with acute mild traumatic brain injury and intracranial hemorrhage

**DOI:** 10.1186/s13017-016-0069-2

**Published:** 2016-03-31

**Authors:** Fu-Yuan Shih, Hsin-Huan Chang, Hung-Chen Wang, Tsung-Han Lee, Yu-Jun Lin, Wei-Che Lin, Wu-Fu Chen, Jih-Tsun Ho, Cheng-Hsien Lu

**Affiliations:** Departments of Neurosurgery, Chang Gung University College of Medicine, Kaohsiung, Taiwan; Departments of Neurology, Chang Gung University College of Medicine, Kaohsiung, Taiwan; Departments of Radiology, Kaohsiung Chang Gung Memorial Hospital and Chang Gung University College of Medicine, Kaohsiung, Taiwan

**Keywords:** Risk factor, Mild traumatic brain injury, Surgical intervention

## Abstract

**Background:**

Mild traumatic brain injury (TBI) patients with initial traumatic intracranial hemorrhage (tICH) and without immediate neuro-surgical intervention require close monitoring of their neurologic status. Progressive hemorrhage and neurologic deterioration may need delayed neuro-surgical intervention. This study aimed to determine the potential risk factors of delayed neuro-surgical intervention in mild TBI patients with tICH on admission.

**Methods:**

Three hundred and forty patients with mild TBI and tICH who did not need immediate neuro-surgical intervention on admission were evaluated retrospectively. Their demographic information, clinical evaluation, laboratory data, and brain CT was reviewed. Delayed neuro-surgical intervention was defined as failure of non-operative management after initial evaluation. Risk factors of delayed neuro-surgical intervention on admission were analyzed.

**Results:**

Delayed neuro-surgical intervention in mild TBI with tICH on initial brain CT accounted for 3.8 % (13/340) of all episodes. Higher WBC concentration, higher initial ISS, epidural hemorrhage (EDH), higher volume of EDH, midline shift, and skull fracture were risk factors of delayed neuro-surgical intervention. The volume of EDH and skull fracture is independent risk factors. One cubic centimeter (cm3) increase in EDH on initial brain CT increased the risk of delayed neurosurgical intervention by 16 % (*p* = 0.011; OR: 1.190, 95 % CI:1.041–1.362).

**Conclusions:**

Mild TBI patients with larger volume of EDH have higher risk of delayed neuro-surgical interventions after neurosurgeon assessment. Longer and closer neurological function monitor and repeated brain image is required for those patients had initial larger EDH. A large-scale, multi-centric trial with a bigger study population should be performed to validate the findings.

## Background

Mild traumatic brain injury is a common presentation at the emergency department. Brain CT is the standard diagnostic tool for detecting the intracranial condition of patients with acute TBI. The incidence of associated intracranial abnormalities is 0.7–20 % [1–4]. Some patients with large intracranial hematoma will undergo surgical management initially following guidelines for the surgical management of traumatic brain injury [5]. Repeat brain CT scans after admission and monitoring the neurologic status [6, 7] are used for the early detection of progressive hematoma and increasing intracranial hypertension (IICP) [6, 8–10] in patients without initially surgical intervention. Surgical intervention is used for evacuation of the hematoma and failure of medical treatment of IICP. Several studies have focused on risk factors of intracranial lesion and delayed hemorrhage and intensive care unit (ICU) monitoring to prevent poor outcome of mild TBI patients [1–4, 6]. Few studies have given attention to the risk of surgical intervention [11] after initial neurosurgeon assessment. Because there is a need for better delineation of potential risk factors and clinical features in this specific subgroup, this study aimed to analyze the clinical features, neuro-imaging findings, and measurements to determine the potential risk factors predictive of surgical intervention in patients with mild TBI and tICH on admission.

## Methods

### Study design

This is a single center retrospective study. Three hundred and forty adult patients (age: 15–75 years) with acute TBI and tICH on initial brain CT admitted within 24 h after onset of acute TBI to Kaohsiung Chang Gung Memorial Hospital, a 2715-bed acute-care teaching medical center in southern Taiwan providing both primary and tertiary referral care, were enrolled. The tICH included epidural hemorrhage (EDH), subdural hemorrhage (SDH), intraparenchymal hemorrhage (IPH), and subarachnoid hemorrhage (SAH). All patients received complete medical and neurologic examinations, and brain CT. Neurosurgeon would be consulted to assessment of neuro-surgical intervention at ER. Neuro-radiologists correlated the neuro-imaging findings. The hospital’s Institutional Review Committee on Human Research approved the study.

The diagnosis of acute TBI was confirmed by clinical history and brain CT. Patients were excluded if they had: 1) penetrating head injury or gunshot wound; 2) moderate-to-severe TBI (Glasgow Coma Score <13); 3) no tICH found on initial brain CT; 4) immediate neuro-surgical intervention on admission; and 5) only chronic intracranial hemorrhage in the initial brain CT.

The criteria for non-operative management [11] were primarily based on the clinical and radiographic findings upon admission, including alert mental status, absent lateralizing signs, basal cistern effacement or obliteration, and midline shift <5 mm. Initial neuro-surgical intervention was defined as an operation done immediately while the patient was at the emergency department. Delayed neuro-surgical intervention was defined as an operation done after the failure of non-operative management.

### Clinical assessment

The patients’ demographic information, mechanism of injury, initial vital signs, Glasgow Coma Score (GCS), complete physical and neurologic examination, laboratory data, and ISS [12] were all assessed. The patients underwent brain CT scan shortly after arriving at the ER. Repeat brain CT scans were performed for any clinical deterioration (e.g., acute-onset focal neurologic deficits, seizures, status epilepticus, or progressively disturbed consciousness) and as routine post-neurosurgical procedure. The principal investigator reviewed all of the initial and follow-up CT scans. In equivocal cases, a second observer made the review. Both were blinded to the laboratory results at the time of clinical and radiologic assessment.

The criteria for TBI-associated early coagulopathy included the presence of thrombocytopenia (platelet count <100,000/ml) and/or elevated international normalized ratio >1.2 and/or prolonged activated partial thromboplastin time >36 s at admission [13, 14]. Early hypotension was defined as systolic blood pressure <90 mmHg and/or diastolic pressure <40 mmHg, documented at the ER [15]. A neurosurgeon evaluated the acute TBI patients and decided on initial neuro-surgical intervention or non-operative management. Neurologic deterioration was defined as a GCS score decline ≥2, acute-onset focal neurologic deficits, seizures, or signs of progressive increased intracranial pressure (IICP). A second brain CT, taken for neurologic deterioration or upon the neurosurgeon’s request, was categorized as improved, worsened, or unchanged, as Sifri mentioned in 2004 [16].

Neurosurgical intervention was defined as placement of craniotomy or craniectomy with or without an intracranial pressure monitor. Patients with intracranial pressure monitor placed were excluded in the neurosurgical group.

### Statistical analysis

Data were expressed as median (inter-quartile range [IQR]). Statistical significance was set at *p* < 0.05. Categorical variables were compared using the Chi-square test or Fisher’s exact test, as appropriate, while continuous variables were assessed by the Mann–Whitney U test. Correlation analysis using the Spearman rank test explored the relationship between age, GCS on admission, and ISS on admission.

Stepwise logistic regression analysis was used to evaluate the relationship between significant variables and therapeutic outcomes, with adjustments made for other potential confounding factors. Variables with zero cell count in a 2-by-2 table were eliminated from logistic analysis and only variables with strong association with poor outcome (*p* < 0.05) were included in the final model. The receiver operating characteristic (ROC) curve analysis was used to estimate an optimal cut-off value for volume of tICH on admission. The areas under the ROC curves were calculated for each parameter and compared. All of the statistical analyses were conducted using the SPSS software package, version 12.0 (Chicago, IL, USA). Statistical significance was set at *p* = 0.05.

## Results

### Baseline characteristics of the study patients

Of the 347 patients with acute mild TBI and tICH admitted, six underwent immediately surgical intervention and one underwent ICP monitor insertion due to orthopedic surgery. Thus, 340 patients with acute mild TBI and tICH were finally included. Based on their characteristics on admission (Table 1), there were 203 males (59.7 %) and 137 females (40.3 %). Their median age was 50 years (range: 16–75 years). Eighteen patients (5.3 %) had an initial GCS score of 13, 66 (19.4 %) had an initial GCS score of 14, and 256 (74.6 %) had an initial GCS score of 15. The most common mechanism of injury was vehicle accident (75.3 %). Hypertension and diabetes mellitus were the two most common underlying diseases. The median ISS was 10 and the median ICU and hospital stay were one and 8 days, respectively.

**Table 1:** Summary of admission characteristics of patients with mild TBI and tICH

Patient demographics	No. of patients
Total patients	340
Median age, years (IQR)	50 (32, 60.75)
Male: female (%)	203 (59.7 %):137 (40.3 %)
GCS score on admission (%)	
13	18 (5.3 %)
14	66 (19.4 %)
15	256 (74.6 %)
Mechanism of injury (%)	
Assault	7 (2 %)
Fall	73 (21.5 %)
Traffic accident	260 (75.3 %)
ISS score on admission (IQR)	10 (9, 16)
Anti-platelet and/or warfarin therapy (%)	13 (3.8 %)
Underlying disease (%)	
Hypertension	91 (26.8 %)
Diabetes mellitus	51 (15.0 %)
Old cerebral vascular accident	9 (2.6 %)
Coronary artery disease	8 (2.4 %)
Arrhythmia	6 (1.8 %)
Liver cirrhosis	5 (1.5 %)
Chronic kidney disease	7 (2.1 %)
Renal failure	5 (1.5 %)
Median ICU stay in days (IQR)	1 (0, 3)
Median hospital stays (IQR)	8 (5,12)

*Abbreviations*: *TBI* traumatic brain injury; *tICH* traumatic intracranial hemorrhage; *GCS* Glasgow Outcome Scale; *ICU* intensive care unit; *IQR* inter-quartile range

### Brain imaging results

The patient characteristics in terms of tICH in the brain CT (Table 2) revealed that 222 (65.3 %) patients had single intracranial hemorrhage. EDH, subarachnoid hemorrhage (SAH), midline shift, skull fracture, and volume of EDH were significantly different between the groups of delayed and no surgical intervention. The median volume of EDH was 30.98 ml higher than volume of SDH and contusive hemorrhage. 6/26 (18.8 %) patients with EDH would undergo delayed surgical intervention. SAH was the most common type of hemorrhage in mild TBI. A few of patients had intra-ventricular hematoma but one third needed delayed surgical intervention. Skull fracture was the most common non-hemorrhagic lesion seen on initial brain CT. Midline shift was highest among patients who underwent delayed surgical intervention.

**Table 2:** Comparison of acute mild TBI with tICH patients who needed delayed neuro-surgical and non-surgical intervention on admission

	Delayed neuro-surgical intervention	Non-neurosurgical intervention	*p* value	OR	95 % CI
	*n* = 13	*n* = 327			
Median age, years, (IQR)	43(25, 49)	50(32, 61)	0.082		
Male/female (%)	9 (69 %)/4 (31 %)	194 (59 %)/133 (41 %)	0.573	0.648	0.196–2.149
GCS at presentation, Median (IQR)	15(14, 15)	15(15, 15)	0.189		
13 (%)	2 (15.4 %)	16 (4.9 %)			
14 (%)	3 (23.1 %)	63 (19.3 %)			
15 (%)	8 (61.5 %)	248 (75.8 %)			
Anti-platelet and/or warfarin therapy	1 (7.7 %)	12 (3.7 %)	0.403	2.188	0.263–18.222
Statin therapy	0	6 (1.8 %)	1.000		
Hypotension	0	4 (1.2 %)	1.000		
Blood test					
WBC count (1000/mL), Median (IQR)	15.00 (12.4, 17.05)	11.7 (8.80, 17.05)	0.023		
RBC count (1000/mL), Median (IQR)	4.74 (4.075, 5.945)	4.59 (4.26, 4.98)	0.401		
Hemoglobin, Median (IQR)	14.10 (12.85, 14.90)	13.60 (12.30, 15.00)	0.606		
Coagulopathy	0	10 (3.1 %)	1.000		
Underlying diseases					
Hypertension (%)	2 (15.4 %)	89 (27.2 %)	0.526	0.484	0.105–2.228
Diabetes mellitus (%)	2 (15.4 %)	49 (15.0 %)	1.000	1.028	0.221–4.780
Old cerebral vascular accident	0	9 (2.8 %)	1.000		
Coronary artery diseases	0	8 (2.4 %)	1.000		
Arrhythmia	0	6 (1.8 %)	1.000		
Liver cirrhosis	0	5 (1.5 %)	1.000		
Chronic renal disease	0	7 (2.1 %)	1.000		
Renal failure	0	5 (1.5 %)	1.000		
ISS score, Median (IQR)	16 (15, 17.5)	10 (9, 16)	0.005		
Single intracranial hemorrhage (%)	6 (46.2 %)	216 (66.1 %)	0.149		
Multiple intracranial hemorrhage (%)	7 (53.8 %)	111 (33.9 %)	0.149		
Type of intracranial hemorrhage					
EDH (%)	6 (46.2 %)	26 (8.0 %)	≤0.001	9.923	3.105–31.708
SDH (%)	6 (46.2 %)	159 (48.6 %)	1.000	0.906	0.298–2.753
IPH (%)	6 (46.2 %)	105 (32.1 %)	0.366	1.812	0.594–5.526
SAH (%)	3 (23.1 %)	178 (54.4 %)	0.044	0.251	0.068–929
IVH (%)	1 (33.4 %)	2 (0.6 %)	0.111	13.542	1.147–159.876
Midline shift (%)	5 (33.4 %)	10 (3.1 %)	≤0.001	19.813	5.495–71.435
Skull fracture (%)	11 (14.3 %)	66 (20.2 %)	≤0.001	21.750	4.707–100.510
Pneumocranium (%)	0	32 (9.8 %)	0.621		
Volume of hemorrhage in initial brain CT					
Volume of EDH, Median (IQR)	30.98 (9.68, 46.86)	2.20 (0.67, 6.71)	≤0.001		
Volume of SDH, Median (IQR)	4.56 (1.13, 17.83)	1.32 (0.15, 5.38)	0.092		
Volume of IPH, Median (IQR)	2.33 (0.11, 7.3)	0.59 (0.11, 2.53)	0.657		

*Abbreviations*: *TBI* traumatic brain injury; *tICH* traumatic intracranial hemorrhage; *N* number of cases; *IQR* inter-quartile range; *OR* odds ratio; *CI* confidence interval; *EDH* epidural hemorrhage; *SDH* subdural hemorrhage; *IPH* intraparenchymal hemorrhage; *SAH* sub-arachnoid hemorrhage; *IVH* intraventricular hemorrhage; *GCS* Glasgow Outcome Scale

### Neurologic deterioration, secondary brain CT, hospital event, and outcome of hospitalization

Recorded hospital events, secondary brain CT, and outcomes (Table 3) demonstrated that 25 patients (7.3 %) had neurologic deterioration that was significantly different between groups with delayed and those without surgical intervention. Fourteen patients (4.1 %) had GCS decline, two (0.6 %) had focal weakness, six (1.8 %) had seizures, and six (1.8 %) had signs of progressive IICP. The decline in GCS and the signs in progressive IICP were significantly different between the delayed and no surgical intervention groups.

**Table 3:** Summary of hospital events, secondary brain computed tomography, and outcome

	Delayed neuro-surgical intervention	Non-surgical intervention	Total (%)	*p* value
	*n* = 13	*n* = 327	*n* = 340	
Neurologic deterioration	12	13	25 (7.3)	≤0.001
GCS decline	7	7	14 (4.1)	≤0.001
Focal weakness	1	1	2 (0.6)	0.075
Seizure	1	5	6 (1.8)	0.210
Progressive IICP signs	4	2	6 (1.8)	≤0.001
Secondary brain CT				
Improved	0	33	33 (19.9)	0.073
Unchanged	3	96	99 (59.6)	0.007
Worsened	10	24	34 (20.5)	≤0.001
Respiratory event	0	7	6 (1.8)	1.000
Cardiac event	0	2	2 (0.6)	1.000
Time of surgical intervention, hours, Mean (IQR)	67.7 (31.5, 125)			
Mean Hospitalization days, Median (IQR)	12 (11, 28)	8 (4, 12)		≤0.001

*Abbreviations*: *GCS* Glasgow Coma Score; *IICP* increased intracranial pressure; *IQR* inter-quartile range; *OR* odds ratio; *CI* confidence interval

In this series, 166 patients received a second brain CT during hospitalization. The result of patients with secondary CT were improved (33/166, 19.9 %), unchanged (99/166, 59.6 %), and worsened (34/166, 20.5 %).

The 13 patients who received delayed neurosurgical intervention included nine with neurologic deterioration and enlarged tICH, three with signs of progressive IICP and unchanged tICH, and one with enlarged tICH on regular brain CT follow-up without neurologic deterioration. The median time of surgical intervention after injury was 67.7 (11.7, 130.9) hours.

Three patients died during hospitalization and the overall mortality rate is 0.9 %. One died due to progressive hemorrhage but the family refused surgery. One died because of pneumonia with severe sepsis and another was due to endocarditis. Seven patients had a respiratory event (2.1 %) and two had a cardiac event (0.6 %). One had respiratory failure with ventilator support and two had myocardial infarction. The mean hospitalization days were 12 an 8 days in patients with delayed neurosurgical intervention and without it.

### Risk factors of surgical intervention in mild TBI

Risk factors of failure of non-operative management in patients with acute mild TBI and tICH (Table 2) were higher WBC count, EDH, larger volume of EDH, midline shift, skull fracture, and higher initial ISS (*p* < 0.05). After stepwise logistic regression, only the volume of EDH was independently associated with delayed neuro-surgical intervention. A cut-off value of 9.3 ml EDH on admission had 83.3 % sensitivity and 84.6 % specificity for predicting delayed neuro-surgical intervention. Furthermore, an increase of one cubic centimeter (cm [3]) in EDH on initial brain CT increased the risk of delayed surgical intervention by 16.0 % (*p* = 0.011; OR: 1.190, 95 % CI: 1.041–1.362). The area under the curve for volume of EDH was 0.917 (95 % CI: 0.797–1.000). Using the ROC curves of the detailed prediction model, for a linear predictor score of 1.67, sensitivity was 83.3 % and specificity was 84.6 % for delayed neuro-surgical intervention. With increased volume of epidural hemorrhage, the sensitivity of the model to detect very positive finding decreased, while the specificity of the model increased. All hematoma >26 ml needed neurosurgical evacuation.

## Discussion

Patient with acute mild TBI constitute a significant number of patients in the emergency department. Regular follow-up brain CT, monitoring in the intensive care unit, and financial difficulties have all been discussed. Surgical intervention is the most important consideration. In the current study, the failure rate of non-operative management is 3.8 % (13/340). Such figures are consistent with those of five recent studies, with a range of 1–8.7 % [6, 10, 17–19]. The rate and number of patients with acute mild TBI undergoing neurosurgical intervention is low. The rate of craniotomy is 0.9–5.8 % and ICP monitor insertion is 0.2–2.9 %. In 2009, Bee et al. reported the largest number and rate of neurosurgical intervention in mild TBI with tICH [6]. Thomas et al. reported the largest number of patient with mild TBI with tICH [10]. Their number of neurosurgical intervention was 18 and 14, respectively. Both studies focused on repeat brain CT, progressive hemorrhage, and ICU monitor. We identified the risk of factors of mild TBI with initial tICH and delayed neurosurgical intervention including volume of EDH.

In the initial brain CT, only volume of EDH was the risk of delayed neuro-surgical intervention. The median volume of EDH was larger than volume of SDH and IPH. Patients can tolerate the larger volume of EDH without neurologic deficit than others of hemorrhage. Chen et al. reported that an EDH volume >30 ml, thickness >15 mm, and midline shift >5 mm tended to require surgery [20]. In this series, there is no successful non-operative management in patients with EDH >26 ml on initial brain CT. All of them had signs of progressive IICP during non-operative management. Moreover, any increase of one cubic centimeter (cm [3]) increased the presence of delayed neurosurgical intervention by 16.0 % (*p* = 0.011; OR: 1.190, 95 % CI: 1.041–1.362). Larger volume of initial EDH tended to have higher risk of delayed neuro-surgical management.

Previous reports found that progressive hemorrhage occurred in approximately 15–28 % of patients with acute mild TBI [6, 16, 21]. Most developed early (within one day after the injury) during the clinical course [22–24]. In this study, 20.5 % of patients had larger hematoma seen in following up brain images. The median time of repeated brain CT is 65.9 (10.1, 128.4) hours in delayed neurosurgical intervention. Most patients (12/13, 92.3 %) had neurologic deficit. The median time of neurologic deterioration and neurosurgical intervention was 51.8 (9.9, 92.9) hours and 67.7 (31.5, 125) hours in delayed neuro-surgical intervention.

In the current study, neurologic deterioration in acute mild TBI accounts for 7.3 % (25/340) of all episodes, including 48 % (12/25) who underwent neuro-surgical intervention. The study by Sifri et al. had 161 patients with mild traumatic brain injury and intracranial bleed on initial cranial CT. Due to abnormal neurologic findings on repeat CT scan, 6 % of these patients underwent emergency craniotomy [17]. In another study, 3.7 % (21/565) had acute neurologic deterioration and 29 % (6/21) led to neuro-surgical or medical intervention [18]. Washington et al. reported 321 patients with mild TBI and abnormal head CT, including four patients (1 %) with neurologic decline and three who underwent neurosurgical intervention [19]. In the present series, there was a higher incidence of neurologic deterioration in the delayed neuro-surgical intervention group.

In terms of laboratory data, higher WBC count is a risk of delayed neurosurgical intervention. Leukocytosis is associated with the severity of injury and outcome [25, 26]. Although the mechanism is still controversial, catecholamines increase the leukocyte count by releasing marginated cells into the circulating pool. Corticosteroids increase the neutrophil count by releasing cells from the storage pool in the bone marrow. In this study, leukocytosis is significantly different in the two groups of non-operative management and delayed neurosurgical intervention. There were thirteen patients received anti-coagulation therapy in our study, but only ten patients had coagulopathy at the time of injury. However, no patient with coagulopathy needs delayed neurosurgical intervention. In our clinical practice, we corrected those patients with coagulopathy as soon as possible after intracranial hemorrhage was diagnosed. Furthermore, we only enrolled patients with mild traumatic brain injury. This could be why no patient with coagulopathy needs delayed neurosurgical intervention in this study.

This study has several limitations. First, the study is retrospective. Second, following brain images during non-operative management are irregular, and that could be a confounding factor. The real rate of progressive intracranial hemorrhage is also unknown. Lastly, there is the short-term follow-up period and relatively small sample size. A larger cohort is necessary to generate more powerful conclusions and to refine predictors of neuro-surgical interventions.

## Conclusions

Larger volume of EDH on initial brain CT had higher risk of delayed neuro-surgical intervention after neurosurgeon assessment. Longer and closer neurological function monitor and repeated brain image is required for those patients had initial larger EDH. A large-scale, multi-centric trial with a bigger study population should be performed to validate the findings.

## Abbreviations

aPTT: activated partial thromboplastin time
BP: blood pressure
ROC: receiver operating characteristic
EDH: epidural hemorrhage
ER: emergency room
GCS: Glasgow Coma Score
ICU: intensive care unit
IICP: increased intracranial pressure
SAH: subarachnoid hemorrhage
SDH: subdural hemorrhage
TBI: traumatic brain injury
tICH: traumatic intracranial hemorrhage
